# Supplementary material for: Generalizing biological surround suppression based on center surround similarity via deep neural network models
Source: PLoS Comput Biol. 2023 Sep 22;19(9):e1011486. doi: 10.1371/journal.pcbi.1011486 (PMC10550176; doi:10.1371/journal.pcbi.1011486)
Supplement: S1 Table — Silent neurons and neurons with too large or small center were excluded from the orientation suppression simulations. But the visualization experiments only excluded neurons with too large or small center and included silent neurons. Silent neurons were defined whose center orientation tuning curve has less than 0.001 variances. Center that is larger than 70% of the theoretical receptive field size was considered too large; smaller than 30% of the theoretical receptive field size was considered too small. (PDF) [file pcbi.1011486.s001.pdf]

Layer	Total	Selected	Silent	Center too large	Center too small
A1	96	5	0	78	13
A2	256	123	0	95	38
A3	384	232	5	60	89
A4	384	199	15	23	154
A5	256	82	31	9	142
V4	128	2	0	120	6
V5	256	85	0	163	8
V6	256	127	0	113	16
V7	256	132	0	53	37
V8	512	271	0	101	91
V9	512	250	0	76	186
V10	512	211	3	62	238
V11	512	255	1	55	202
V12	512	254	4	52	203
V13	512	297	3	29	183
