# Supplementary figures and images for: Generalizing biological surround suppression based on center surround similarity via deep neural network models
Source: PLoS Comput Biol. 2023 Sep 22;19(9):e1011486. doi: 10.1371/journal.pcbi.1011486 (PMC10550176; doi:10.1371/journal.pcbi.1011486)

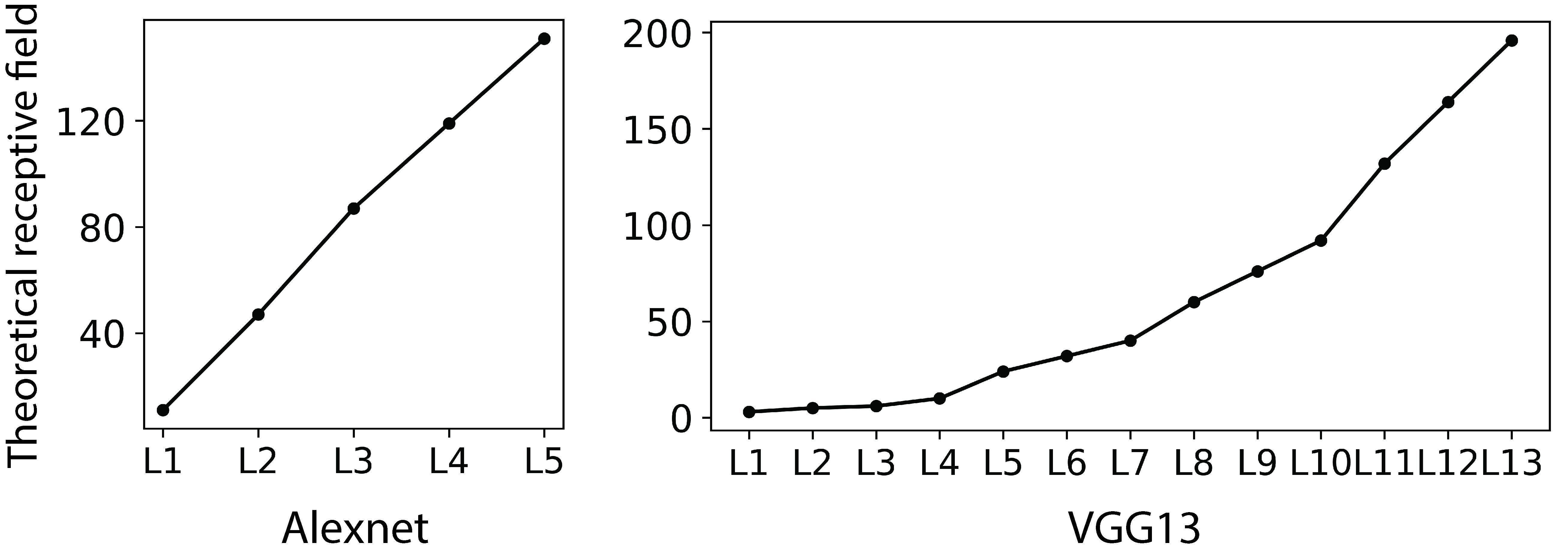

Supplement: S1 Fig — See Methods for more details. (TIF) [file pcbi.1011486.s002.tif]

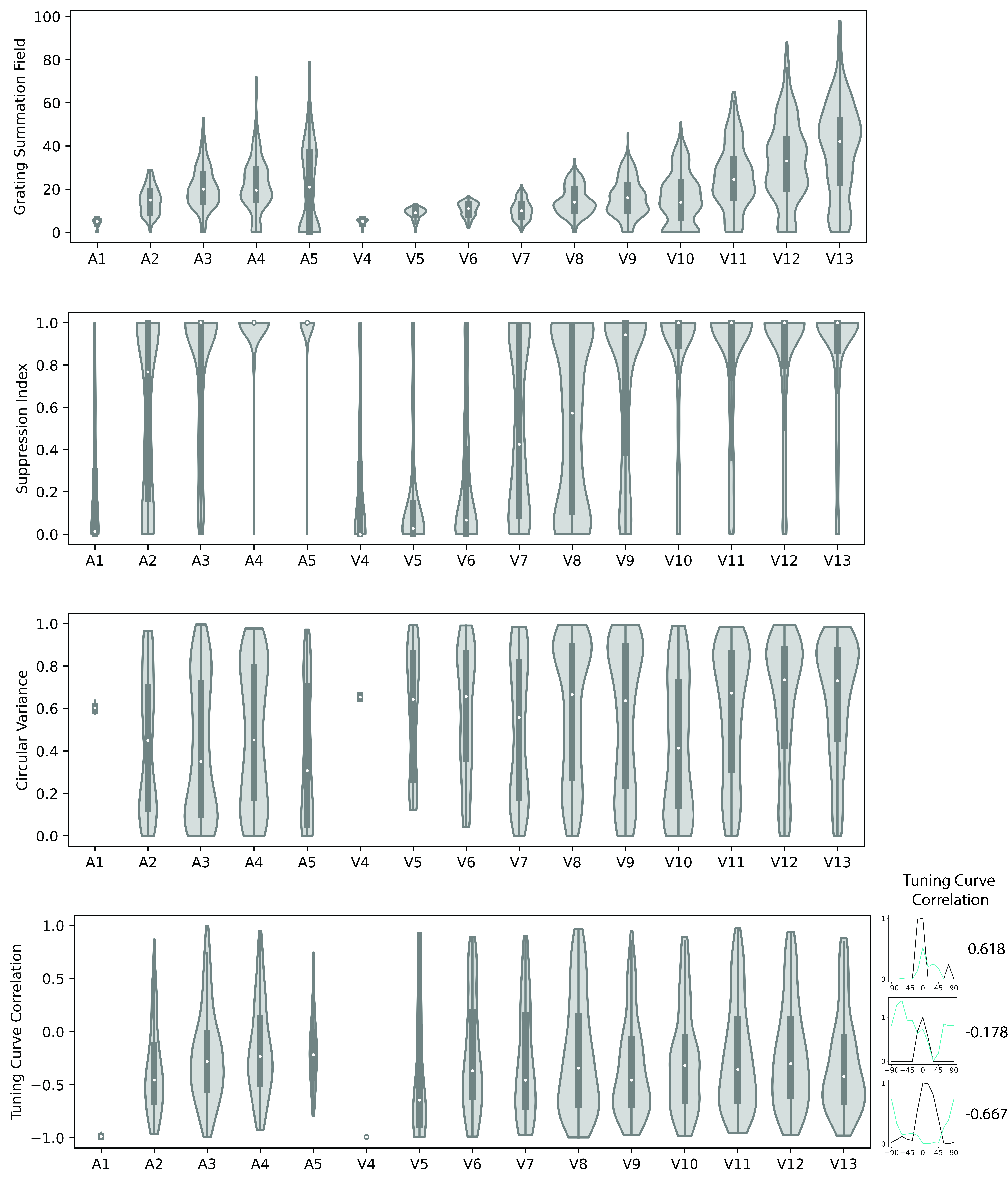

Supplement: S3 Fig — Grating summation field is defined by the smallest diameter that elicits at least 95% of the maximum response. Suppression Index is a metric of surround suppression with grating stimuli which is defined as 1—(suppressed response / peak response). Circular variance is a metric that characterizes orientation selectivity and is defined in [65]. Small values indicate high orientation selectivity; large values indicate low orientation selectivity. Tuning curve correlation is the Pearson correlation coefficient between the center orientation tuning curve and the surround suppression tuning curve. Correlation coefficients are all negative on average in all layers, which indicates the most suppressive surround orientation matches the optimal center orientation. Three example neurons with different turning curve correlations are shown on the right. (TIF) [file pcbi.1011486.s004.tif]

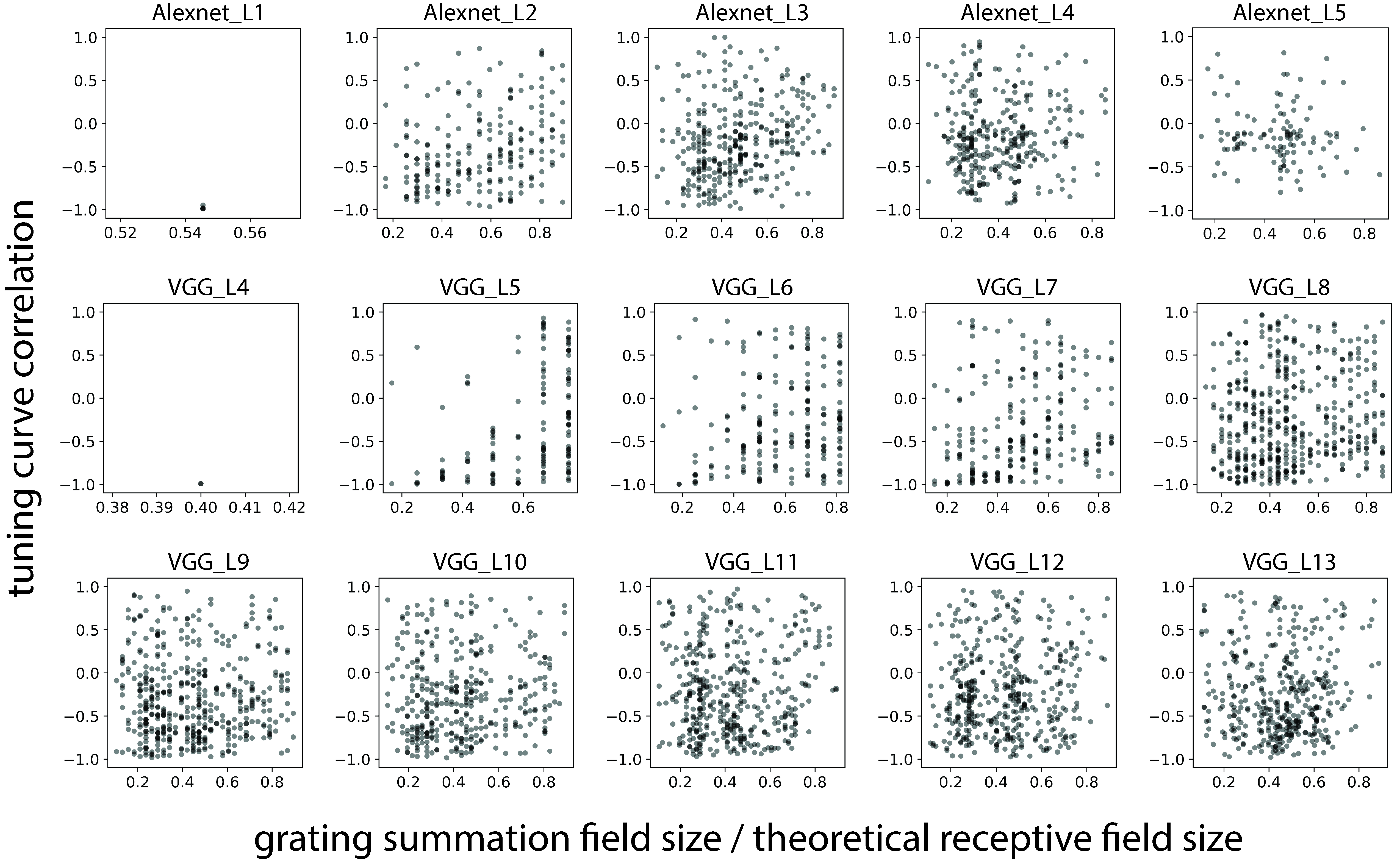

Supplement: S4 Fig — In the main experiments, we focused our analysis on the neurons with sufficiently large center and surround sizes. In some layers, especially middle layers, neurons with negative tuning curve correlation are concentrated at a center-surround ratio of 0.2 to 0.5. (TIF) [file pcbi.1011486.s005.tif]

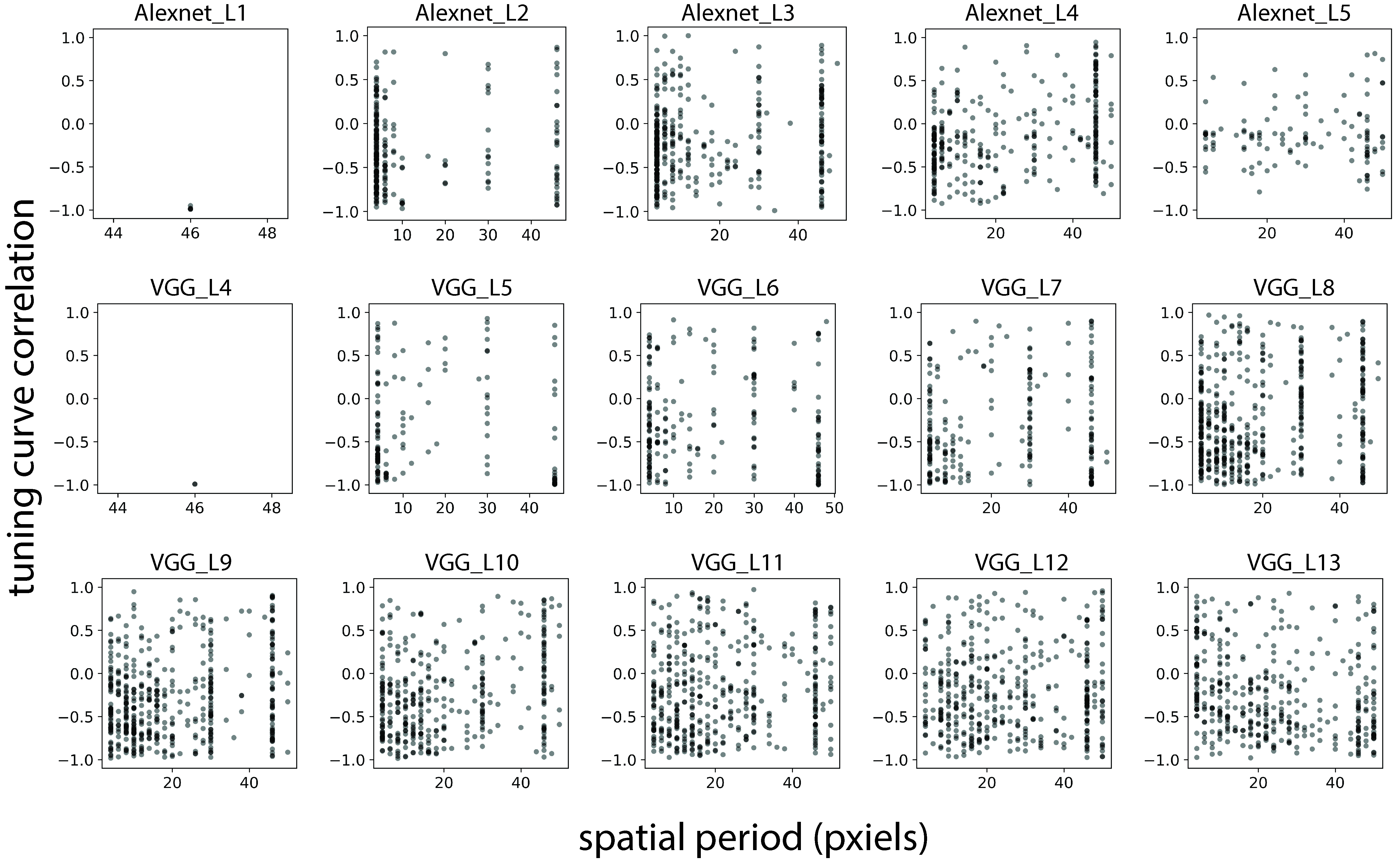

Supplement: S5 Fig — We found the distributions are multi-modal. Many neurons with negative tuning curve correlation are concentrated below 30-pixel spatial period. There are some neurons that have large spatial periods, e.g. 50 pixels. Those neurons are likely to be tuned to large color patches or complex patches beyond simple gratings. (TIF) [file pcbi.1011486.s006.tif]

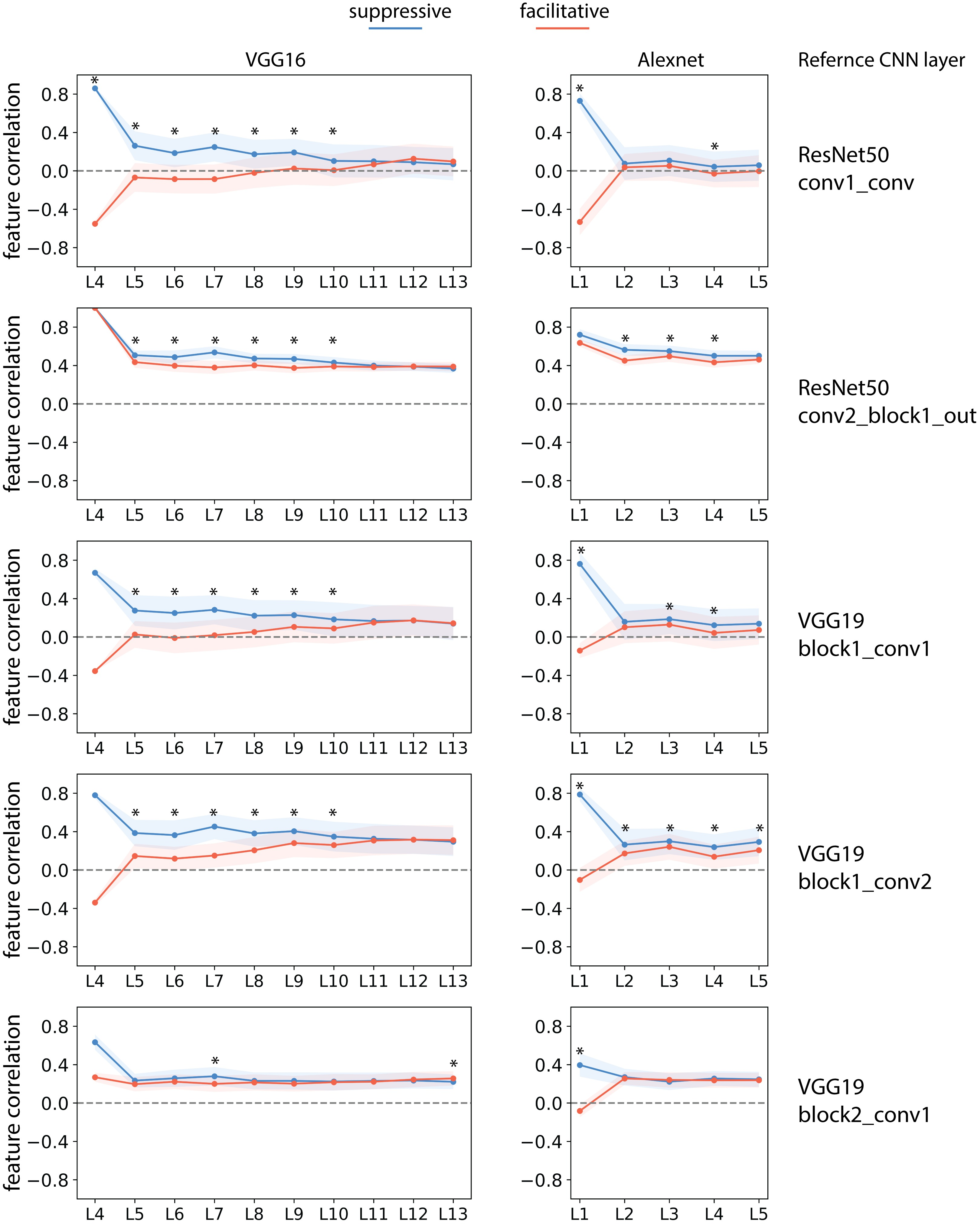

Supplement: S6 Fig — Feature correlation of the center and surround depends on the choice of the reference feature maps. Though the absolute value varies with the feature map choice, they share similar trends that the most suppressive surround has higher feature correlations than the most facilitative surround in early/middle layers in VGG16. Note that a proper choice of feature map should not be a layer that is too deep and has large receptive fields, in which case the surround feature also “sees” the center. For details on how to compute feature correlation, see Fig 3 caption. The shaded area indicates standard deviation. Asterisks indicate p value smaller than 0.05 in paired t-test. (TIF) [file pcbi.1011486.s007.tif]

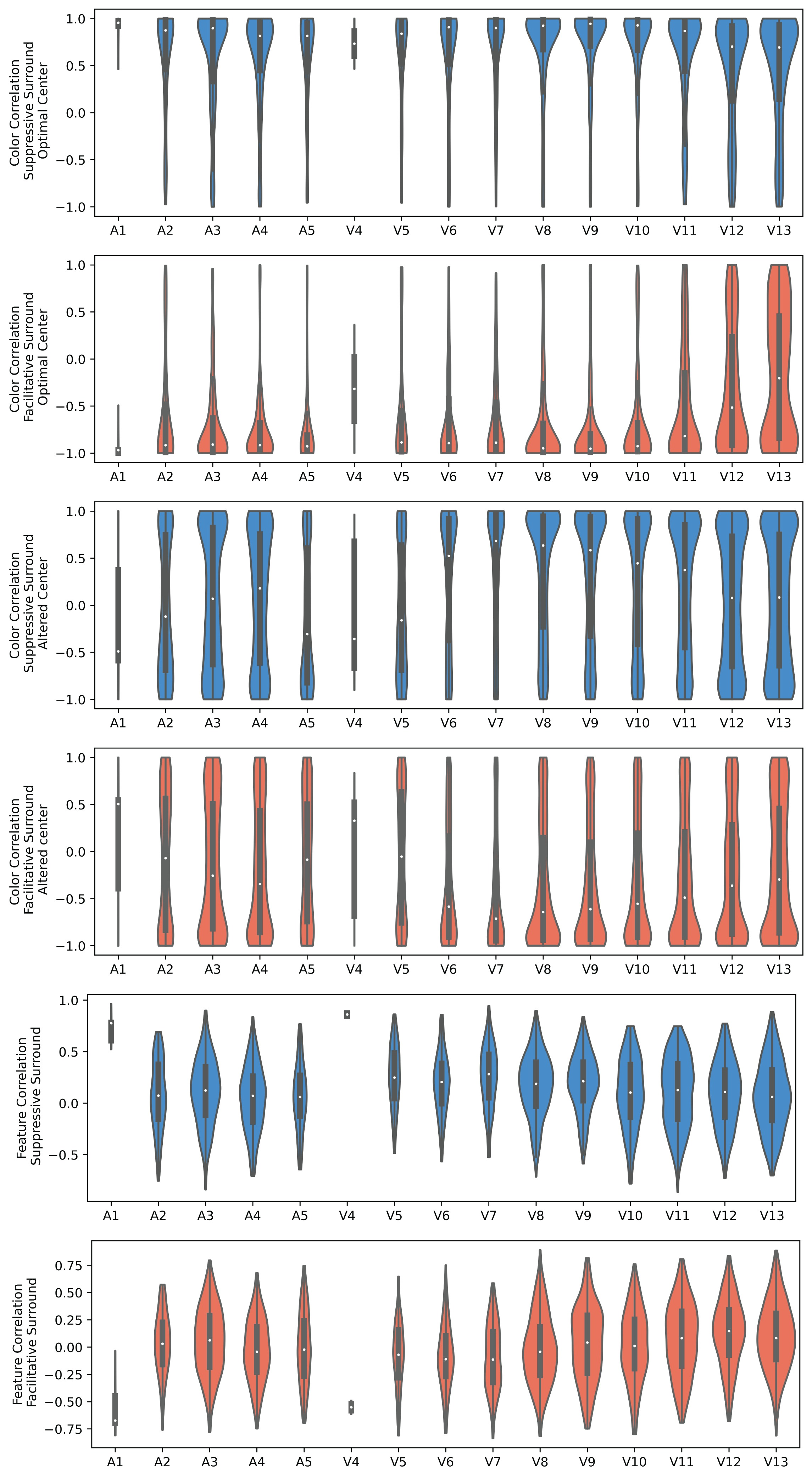

Supplement: S7 Fig — Same measure as in Figs 3 and 5 but plotted in violin style to show population distribution. (TIF) [file pcbi.1011486.s008.tif]

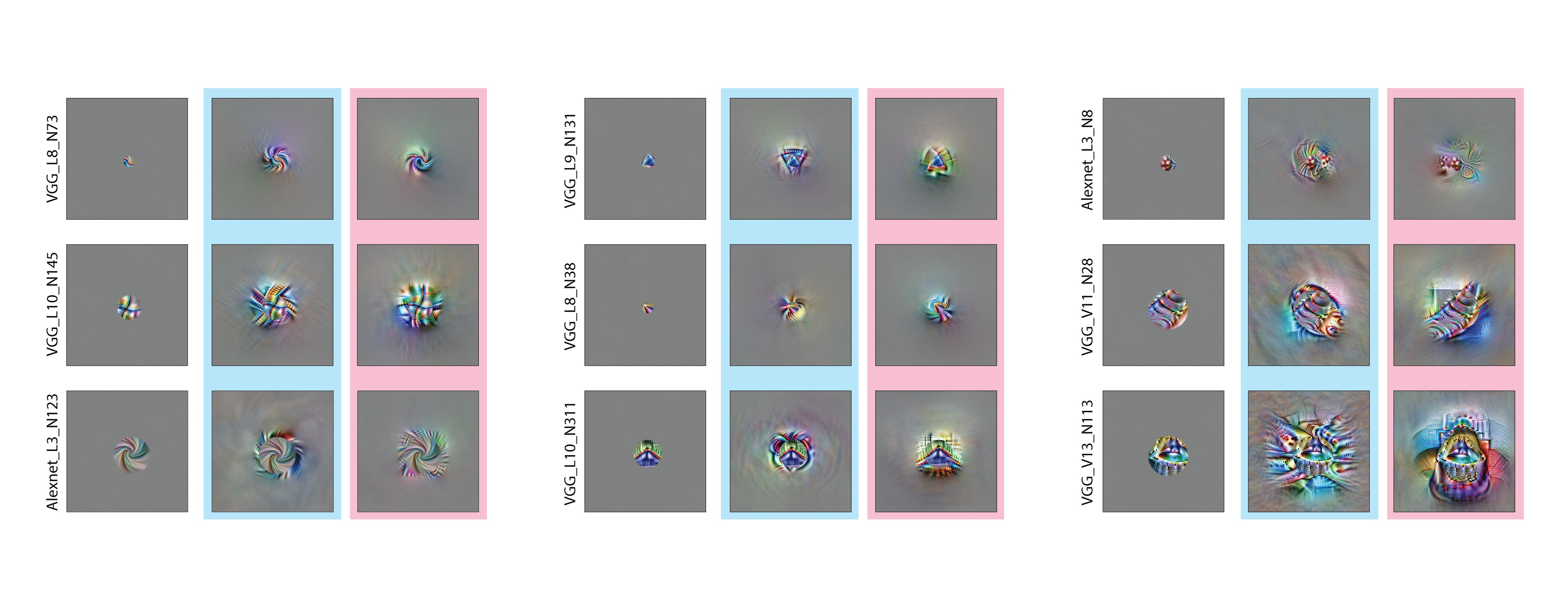

Supplement: S8 Fig — The blue background denotes the most suppressive surround; the pink background denotes the most facilitative surround. These neurons do not have clear center-surround contrastive features; they are likely to include surround features that are geometrically arranged rather than uniform across the surround or features that are arranged as object-like shapes. (TIF) [file pcbi.1011486.s009.tif]

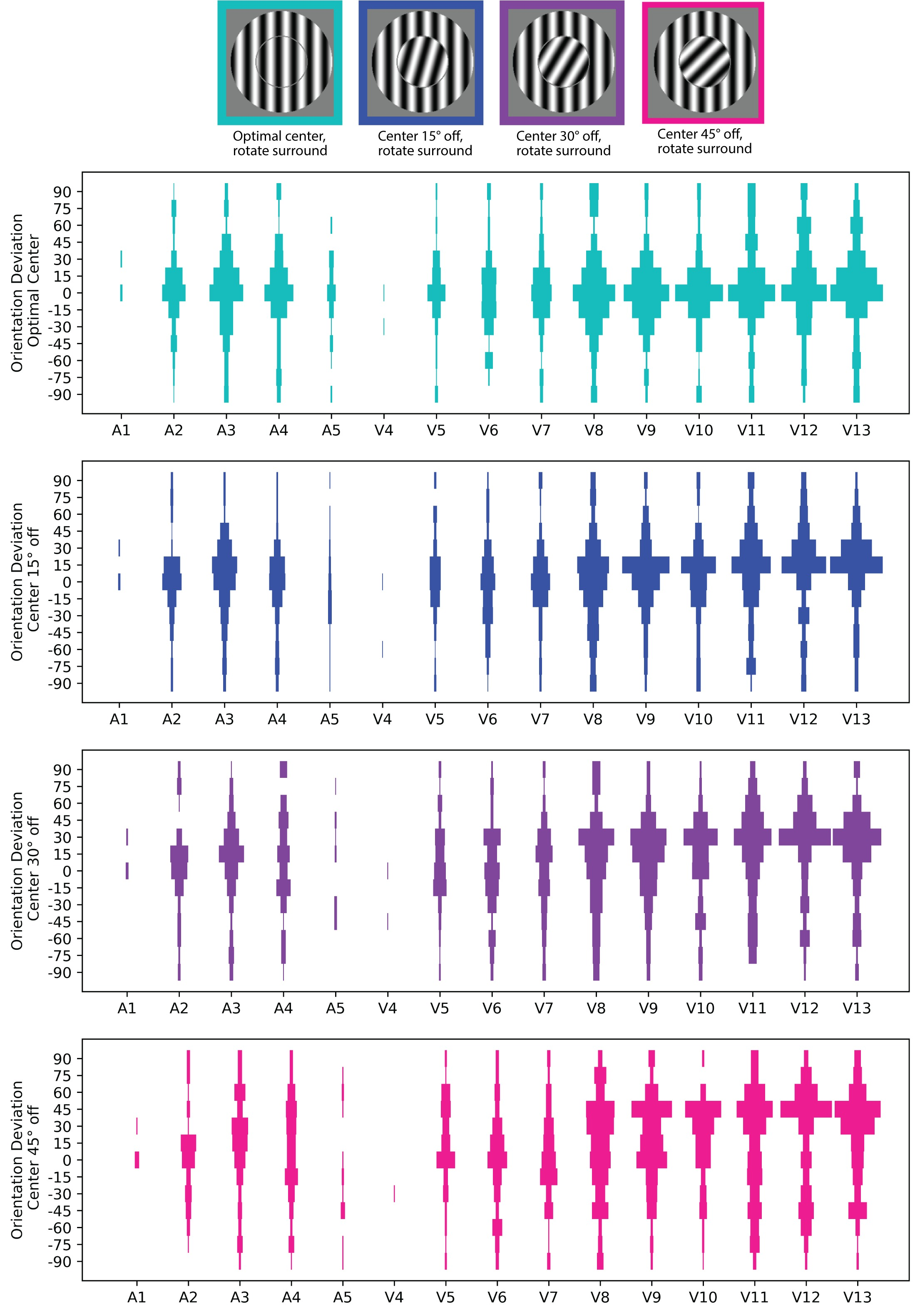

Supplement: S9 Fig — Orientation deviation is defined as the difference between the most suppressive surround orientation and the optimal orientation. To plot this histogram, we included another selection criterion that the surround suppression tuning curve must have at least 0.001 variation, because if a surround suppression tuning curve is flat, there is not a meaningful orientation that has maximum suppression; therefore, it is not informative including in the histogram. In later layers (layer 8 and beyond) in VGG16, orientation deviations match the center orientation better on average. (TIF) [file pcbi.1011486.s010.tif]

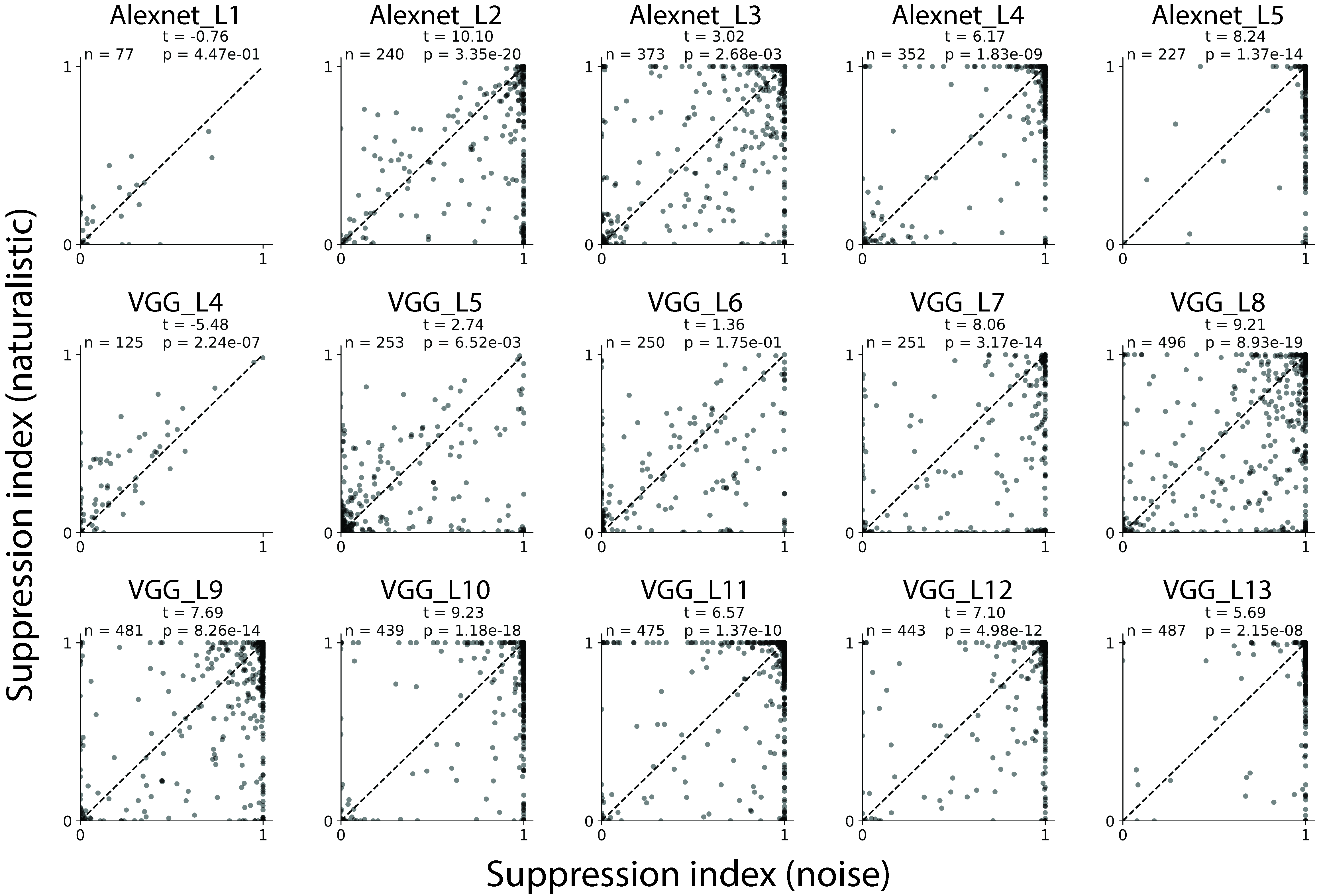

Supplement: S10 Fig — Each dot represents a neuron. In most middle and later layers, neurons have higher suppression indexes with noise images than with naturalistic images, as indicated by the positive t-value and small p value. T and P values are from paired t-test. (TIF) [file pcbi.1011486.s011.tif]

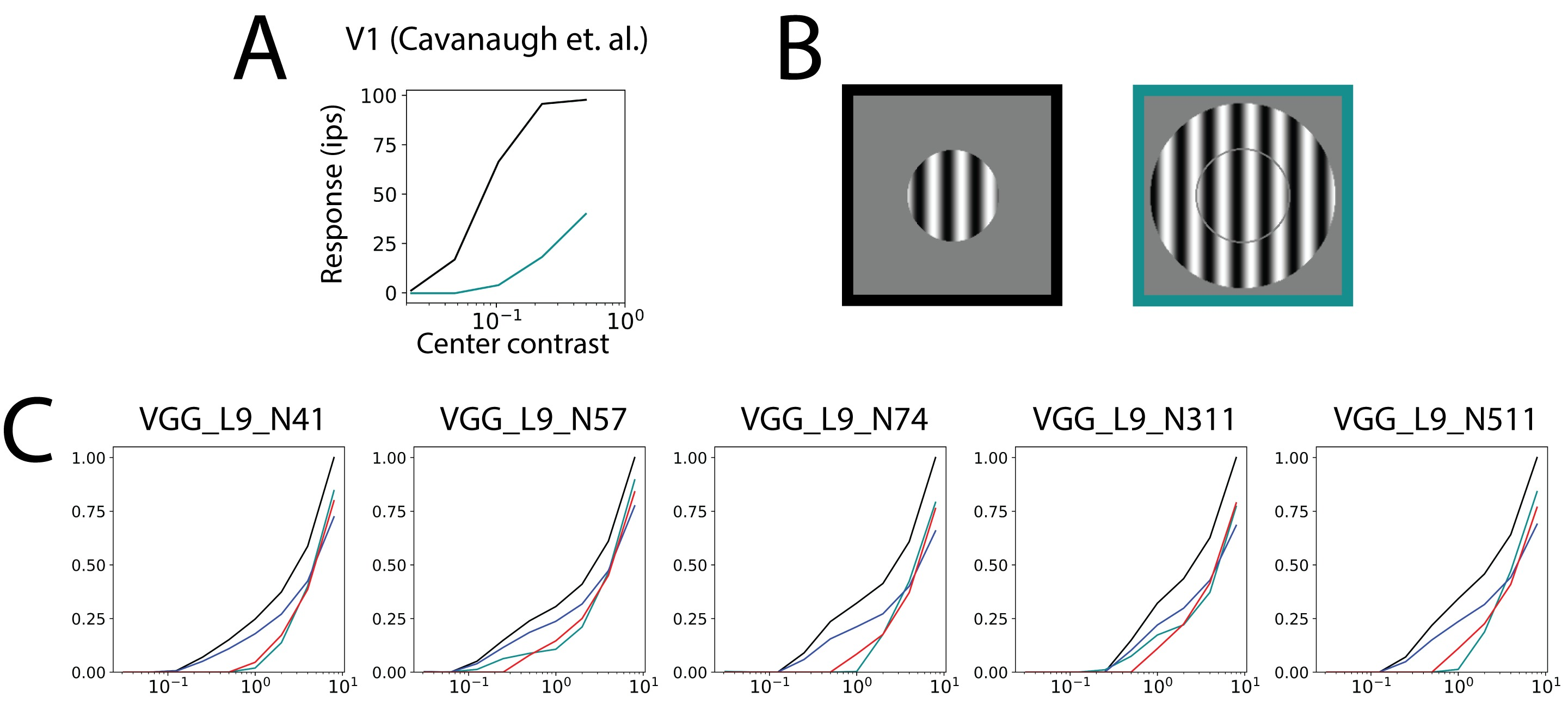

Supplement: S13 Fig — Contrast values were normalized to the regular pixel value range. We fixed the surround contrast at 1, changed the center contrast, and measured the contrast response function. We then fitted two curves with subtractive and divisive models. The subtractive model is described as Rs = max(0, Rc—a), where Rc is the responses of the center stimuli; Rc is the responses of the center stimuli with preferred surround; a is a subtractive parameter that is to be fitted. The divisive model is described as Rs = Rc/b, where b is a divisive parameter that is to be fitted. A. An example V1 neuron from a reference neurophysiology study (reproduced from [16]). Black line denotes no surround; cyan line denotes orthogonal surround. The contrast responses are shifted rightward and downward with surround suppression. B. Stimuli examples used in the experiments. C. Example CNN neurons with different behaviors. Blue line denotes a fitted surround suppression contrast curve with the divisive model; red line denotes a fitted surround suppression contrast curve with the subtractive model. (TIF) [file pcbi.1011486.s014.tif]

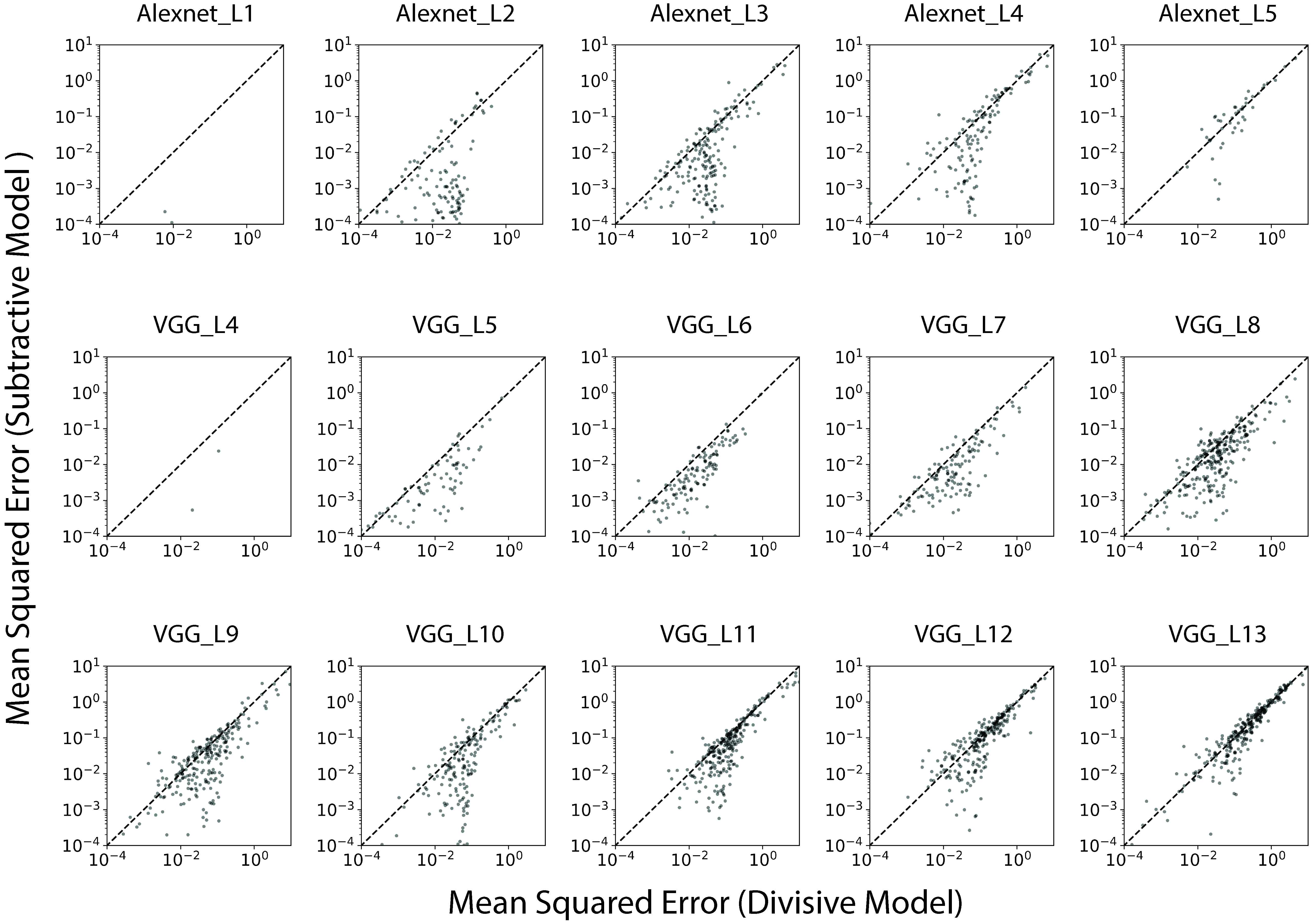

Supplement: S14 Fig — To determine if the surround suppression effect is more likely to be in a subtractive form or divisive form, we fitted contrast curves in S9 Fig with no surround and preferred surround by two models. The x-axis is the fitting error (mean squared error in log scale) of the divisive model; The y-axis is the fitting error of the subtractive model. Points below the diagonal line indicate neuron’s surround is more likely to be subtractive than divisive, which is commonly seen in most layers, especially early layers in both networks. (TIF) [file pcbi.1011486.s015.tif]

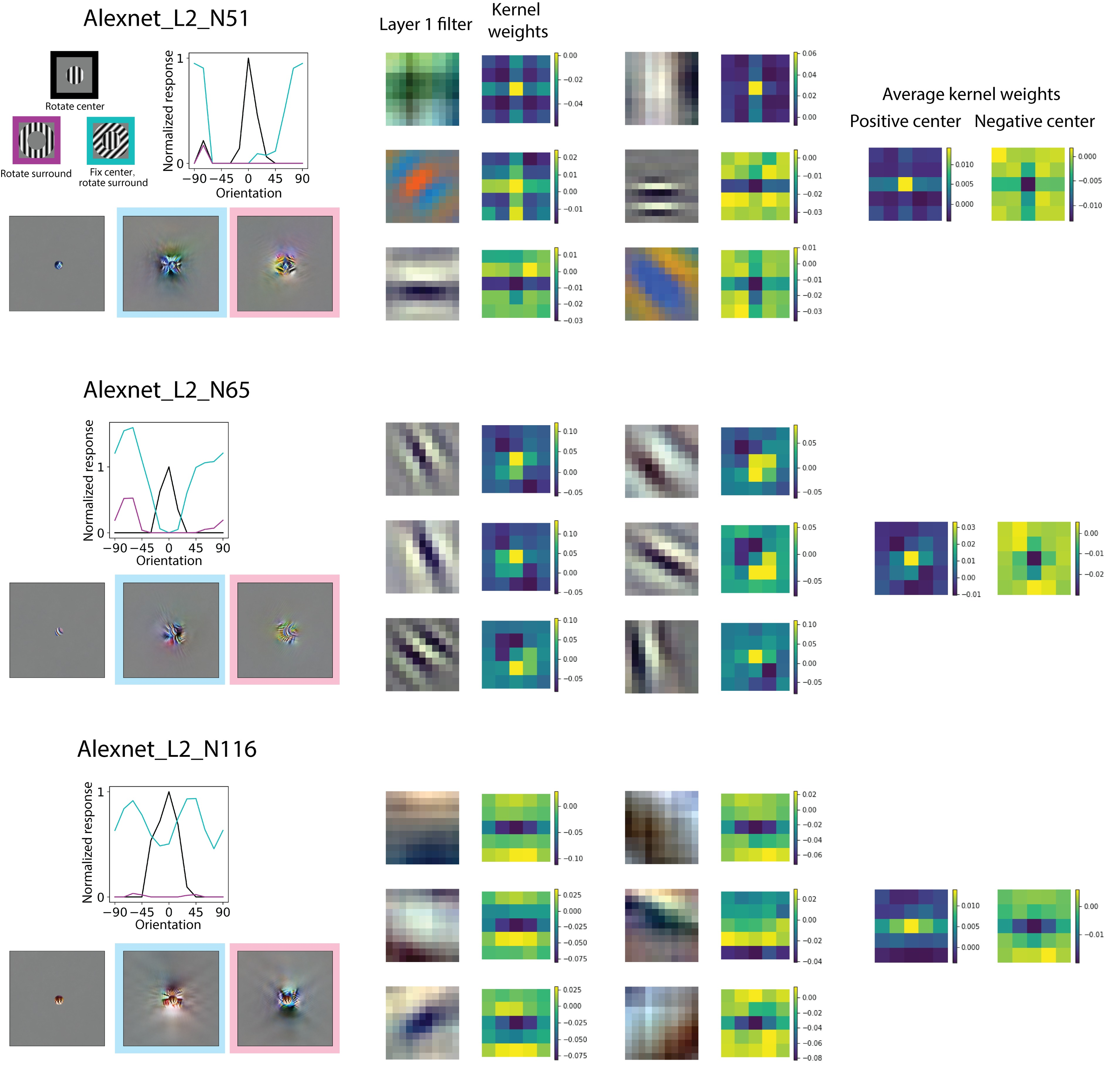

Supplement: S15 Fig — The homogeneous suppressive surround could be implemented by the center surround kernel weights structure. A subtractive form of surround suppression can be seen as the kernel weights having different signs in the center and surround. We show three example neurons in Alexnet layer 2. From left to right: tuning curves and visualization of the example neurons, six most contributing first-layer filters and corresponding kernel weights to the example neurons, and averaged kernel weights of kernels with positive or negative center weight. The most contributing first-layer filters are found by sorting the sum of absolute values of kernel weights. Most of the kernels have a center-surround structure. This indicates a subtractive form of surround suppression. (TIF) [file pcbi.1011486.s016.tif]

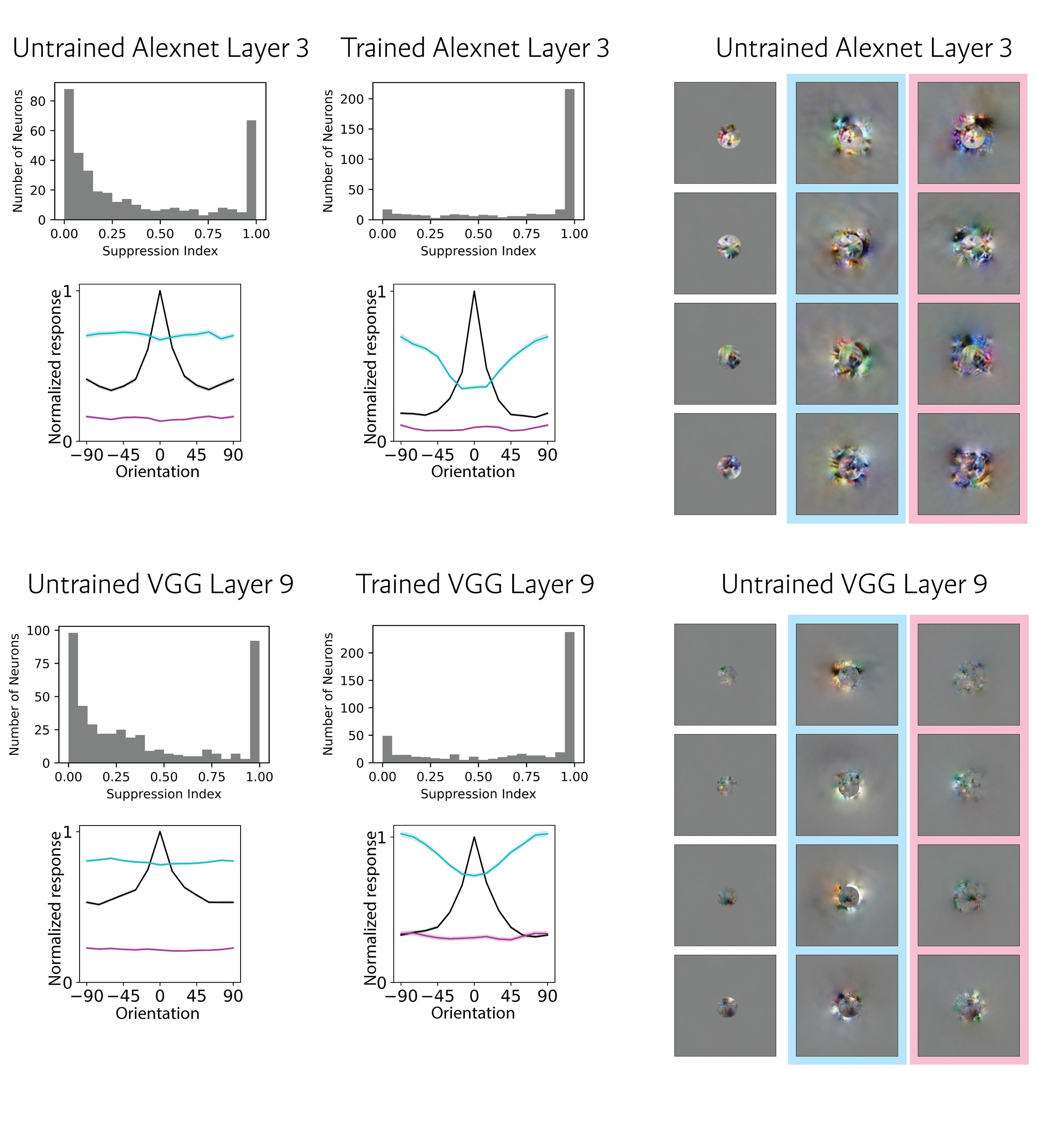

Supplement: S16 Fig — Suppression index, surround suppression curves and visualizations are shown for untrained Alexnet layer 3 and untrained VGG16 layer 9. While most neurons in trained networks showed close to 1 suppression index (strong surround suppression), neurons in untrained networks had bimodal distribution with the majority having close to 0 suppression index (no surround suppression). This was also reflected in the surround suppression curves (cyan), which in untrained networks were flat. On the right is the visualization of several example neurons in the untrained networks. There was no effects like most suppressive surround matches center. All the visualizations look like natural spectrum noise with no high-order textural appearance. (TIF) [file pcbi.1011486.s017.tif]
